# Manipulation of Plant Growth Regulators on Phytochemical Constituents and DNA Protection Potential of the Medicinal Plant* Arnebia benthamii*

**DOI:** 10.1155/2018/6870139

**Published:** 2018-01-04

**Authors:** Javid A. Parray, Azra N. Kamili, Sumira Jan, Mohammad Yaseen Mir, Nowsheen Shameem, Bashir A. Ganai, Elsayed Fathi Abd_Allah, Abeer Hashem, Abdulaziz A. Alqarawi

**Affiliations:** ^1^Department of Environmental Science, Govt. SAM Degree College Budgam, Jammu and Kashmir 191111, India; ^2^Centre of Research for Development, University of Kashmir, Srinagar, Jammu and Kashmir 190006, India; ^3^ICAR-CITH, Rangreth, Srinagar, Jammu and Kashmir 190003, India; ^4^Plant Production Department, College of Food and Agricultural Sciences, King Saud University, P.O. Box 2460, Riyadh 11451, Saudi Arabia; ^5^Botany and Microbiology Department, College of Science, King Saud University, P.O. Box 2460, Riyadh 11451, Saudi Arabia

## Abstract

*Arnebia benthamii* of the family Boraginaceae is a critically endangered nonendemic plant of the Kashmir Himalayas and is used to treat a number of human diseases. The current study was based on developing an* in vitro* micropropagation protocol vis-à-vis induction of various secondary metabolites under* in vitro* conditions for the possible biological activity. A tissue culture protocol was developed for* A. benthamii *for the first time in the Himalayan region using varied combinations and proper media formulations, including various adjuvants: Murashige and Skoog (MS) media, growth hormones, sugars, agar, and so forth. The influence of different media combinations was estimated, and the MS + thidiazuron (TDZ) + indole 3-acetic acid (IAA) combination favors a higher regeneration potential. The higher amounts of chemical constituents were also recorded on the same treatment. The* in vitro* plant samples also showed a noteworthy effect of scavenging of hydroxyl radicals vis-à-vis protection from oxidative DNA damage. The* in vitro* raised plants are good candidates for the development of antioxidant molecules.

## 1. Introduction

Medicinal plants are vital curative agents for curing human ailments. The overexploitation of natural plant resources encouraged various programs such as conservation, micropropagation, and incremental plant architecture [1, 2]. Looking at the present scenario, a significant proportion of the Northwest Himalayan (NWH) medicinal herbs, particularly* Arnebia benthamii *(Wall. ex G. Don) Johnston [syn.* Macrotomia benthamii* (Wall.) DC.], have been rendered rare and threatened over the past few years because the demand for various plant products has increased. Even though different initiatives such as habitat protection as well as Seed and Field gene banks have been started, these techniques may still prove inadequate. The tissue culture technique has become established as a very valuable methodology among the different conservation and multiplication practices presently being employed for increasing the number and improving the development of numerous economically important and threatened medicinal plants [2–5].* A*.* benthamii* (Wall. ex G. Don) Johnston [syn.* Macrotomia benthamii* (Wall.) DC.] of the family Boraginaceae, locally known as Kahzaban, is a perennial medicinal herb growing in the subalpine and alpine zones of the Northwest Himalayas [6]. It is an important medicinal plant, prominent upon the list of 59 medicinal herbs that have been prioritized for conservation in NWH [6] due to their high extinction threat [7]. It is an alpine herb occurring at an altitude of 3500–4000 m, from Kashmir to Western Nepal, normally on open slopes with stony or rocky substrates. In Kashmir Valley, it is confined to certain areas such as Sinthon Top, Duksum, Karnah, Gurez, Lolab, Sonamarg, and Kargil and is rarely found [8, 9]. It is classified as a critically endangered nonendemic plant of Kashmir [10, 11].* A. benthamii *has been listed in the Indian Red Data Book due to its overexploitation for various purposes [12, 13]. Numerous secondary metabolites (i.e., shikonin and its intermediates, alkannins, other naphthoquinones, etc.) have been reported from* Arnebia *spp. However, “Gule Khazaban,” a very expensive medicine, is derived from* A. benthamii* [11, 13, 14].* Arnebia* spp. are reported to be traditional ayurvedic medicines for the treatment of diseases of the throat and tongue, fevers, and cardiac symptoms. The flowers are known to have a comforting effect on heart patients [11], while its roots have antiseptic and antibiotic properties [9]. Conservation of the existing germplasm of such a useful and threatened species is, therefore, a strategic need. Harborne and Baxter [15] have documented the anticancer properties of Arnebin 1 and Arnebin 3 isolated from the* Arnebia* species, and anti-HIV activity has been reported in* Arnebia euchroma *extracts [16].

Due to the higher incidence of microbial pathogens in foods and to control the antibiotic resistant bacterial strains by novel plant extracts [17, 18], search should be continued to propagate medicinal plants by tissue culture biotechnology and to purify their medicinally active compounds for development of a novel treatment of pathogens [19]. Consequently, the study employed herein was undertaken to develop a tissue culture protocol for micropropagation of the depleted germplasm of* A. benthamii. *The study also endeavors to establish the effect of plant growth hormones on the induction of chemical constituents and antioxidant potential of* in vitro* raised plants.

## 2. Materials and Methods

### 2.1. Chemicals Required

MS medium, plant growth hormones (BAP/IAA/TDZ/IAA/NAA), mercuric chloride, agar, and solvents such as methanol (HPLC), water (HPLC), and ethyl acetate (HPLC) were purchased from* HiMedia*,* Merck,* and others.

### 2.2. Plant Collection and Authentication

The whole plants with roots and seeds of* A. benthamii* were collected from Sinthon Top of the Kashmir region (India) (3748 m a.s.l.). The plants were collected in the months from July to September 2013. The plants were authenticated at Kashmir University Herbarium, Centre of Plant Taxonomy, with accession number 1748.

### 2.3. Sterilization of Seeds and Media Selection

Seeds were used as experimental material. Seeds soaked overnight were washed with a few drops of laboratory detergent (Labolene) and 2-3 drops of Tween 20 (surfactant) after washing under running tap water. Chemical sterilization of the seeds was achieved by treating them with 0.1% streptomycin (20 min) followed by 0.1% HgCl_2_ (5 min) and 70% ethanol (45 sec). The seeds were washed 3-4 times with autoclaved double-distilled water to remove all traces of sterilant before inoculating on Murashige and Skoog (MS) basal medium [20] containing 3% (w/v) sucrose* (HiMedia)* and 0.8% (w/v) Difco Bacto Agar* (HiMedia)*. Different plant growth hormones such as thidiazuron (TDZ), indole 3-acetic acid (IAA), kinetin (Kn), 6-benzylamino purine (BAP), and indole 3-butyric acid (IBA) were augmented with MS medium for various morphogenetic responses. The media were sterilized at 121°C for 20 min, and the pH of the medium was adjusted to approximately 5.5. The cultures were incubated in a culture room maintained at 25 ± 3°C with relative humidity of 60–70%. The whole plantlets after cleaning with sterile distilled water were transplanted into pots containing peat-vermiculite-sand-soil mixture (ratio 1 : 1 : 1 : 1, v/v). All the experiments were done in RBD manner. The number of shoots and their length per plant were recorded.

### 2.4. Analysis of Various Chemical Constituents

In this trial, the four best plant hormonal combinations were selected based on higher shootlet formation. The four selected treatments were assigned as T1, T2, T3, and T4 and were compared with plants (T0) collected from the natural habitat. Different metabolites were analyzed.

### 2.5. Alkaloids

The plant samples obtained from different cultural conditions were dried and macerated in a grinder to a powder form and 2.5 g of each plant sample was extracted with ethanol (200 ml) with 20% acetic acid added for 4 h. The filtrate was concentrated to approximately 25 ml and conc. NH_4_OH was added for precipitate formation. The precipitate was further washed with dilute NH_4_OH. The precipitate was dried and weighed, and the amount of alkaloids was determined. The filtrate was dried and weighed [21, 22].

### 2.6. Phenolics

The plant sample powder (1 g) was extracted with 80% ethanol (20 ml). The supernatant obtained was evaporated to dryness and was resuspended in 5 ml of H_2_O. Folin's reagent (0.5 ml) was added to different aliquots (0.1–1 ml), followed by Na_2_CO_3_ (2 ml). A final volume up to 5 ml was obtained with dist. water. The tubes were kept in a water bath for 1 min and slightly vortexed. The reading was taken at 650 nm with standard catechol (2 mg%) [23].

### 2.7. Tannins

The plant sample (2 g) of plant powder was extracted 3 times with acetone (acetone 70%). The supernatant taken was diluted to 3 ml by dist. water. Then, different components in 0.1 M HCl (i.e., 0.016 M K_3_ [Fe(CN)_6_] (1 ml) and 0.02 M FeCl_3_ (1 ml)) were added. The tubes were kept for 15 min after vortexing. The stabilizer solution (5 ml water, H_3_PO_4_, and 1% gum arabic [3 : 1 : 1]) was added, and the solution was revortexed. The reading was taken at 700 nm, and the concentration was determined against gallic acid (1.9 mg%) [24].

### 2.8. Total Flavonoid Content

0.5 ml of the supernatant from the plant sample solution (2 mg/2 ml) was mixed with water (2 ml) and then 5% NaNO_2_ solution (0.15 ml) was added. Then, 10% AlCl_3_ solution (0.15 ml) was added and allowed to stand for 6 min followed by 4% NaOH solution (2 ml), and the final volume was made up to 5 ml with water and allowed to stand for 15 min. Absorbance was checked at 510 nm versus a water blank [25].

### 2.9. DNA Damage Assay

The antioxidant potential was determined by observing the DNA protection ability of the* in vitro* plant samples [14]. 15 *μ*l of calf thymus DNA (25 mg%) was dissolved in 20.0 mM phosphate buffer saline (pH 7.4), and to this solution, different concentrations of plant extracts were added and the mixture was incubated for 15 min at room temperature. Then, the oxidative substances (i.e., 1 *μ*l of 30 mM H_2_O, 1 *μ*l of 20 mM ferric nitrate, and 1 *μ*l of 100 mM ascorbic acid) were added, and the whole reaction was held for 1 h at 37°C. The reaction was stopped by the addition of a loading dye (40% sucrose and 0.25% bromophenol blue). Gel electrophoresis (0.7% agarose/TAE buffer) was run at 100 V for observing changes in DNA and was visualized by the Gel Doc system (Labnet, Germany).

### 2.10. Statistical Analysis

The whole data set was subjected to ANOVA by SPSS software, and significance was set at *P* < 0.005.

## 3. Results and Discussion

### 3.1. Seed Germination and Shootlet Formation

**Figure 1:**
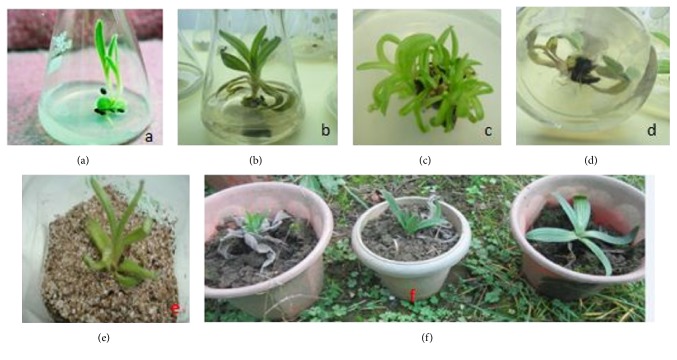
*In vitro *culture of* Arnebia benthamii *Wall. (a) seed germination on 1/2-MS + 5 *μ*M BA; (b) shootlet elongation on 20 *μ*M BA; (c) shootlet formation on 4 *μ*M BA + 1 *μ*M IBA; (d) root formation on 7 *μ*M BA; (e) plantlets in a 1 : 1 mixture of autoclaved sand : soil; (f) plantlets in net house.

The sterilized seeds were inoculated on MS full- and half-strength medium supplemented with gibberellic acid GA_3_ (1.5–20 *μ*M). Complete germination with elongated shoots was observed on 1/2 MS + GA_3_ (15 *μ*M). The minimal time period required for seed germination initiation was recorded as 20 days with 78% seed germination response to the same treatment (Table 1 and Figure 1(a)). Embryonic shoot tips were excised and further cultured on a multiplication medium using auxin/cytokinin combinations. The cytokinins BAP and TDZ (1.5–10.5 *μ*M) were used separately in combination with either IAA or NAA (in the conc. range of 1–10 *μ*M) on MS ×1/2 medium for the multiplication of shoots. The concentration of BAP (4.5 *μ*M) and IAA (4 *μ*M) resulted in the average number of multiple shoots (9.1) exhibiting slightly blackish basal portion. With a further increase in concentration, the number of shoots showed a decreasing trend and the lowest number of shoots was noticed. In another trial, the higher average number of multiple shoots (11) with broad and healthy shoots was obtained on MS 1/2 + BAP (5.5 *μ*M) + NAA (5 *μ*M) (Figures 1(b) and 1(c)). Shoot tips of* A. benthamii* cultured on MS ×1/2 basal medium supplemented with different concentrations of TDZ and IAA/NAA resulted in multiple shoot regeneration with a friable brownish callus at the basal zone of the plant tissues, which was quite significant compared with previous trials. The best response was scored with shoot tips on 3 *μ*M of TDZ and 1.5 *μ*M of IAA which resulted in an average of 20.1 multiple shoots with slight friable callus formation, and with a further increase in concentrations up to 10 *μ*M, the shoot number was found to decrease (Table 2). In another trial, shoot tips cultured with different concentrations of TDZ were used in combination with NAA (0.5–10.0 *μ*M) for multiplication of shoots and the maximum average number of shoots (15.3) with 90% shooting response on MS 1/2 + TDZ (5.0) + NAA (2.5) with low friable brownish callus formation at the basal end. However, the response to the multiple shootlet formation was lower compared to the TDZ/IAA combination.

Different concentrations of IAA/NAA used along with BAP/TDZ resulted in multiple shoot regeneration and further increase in concentration, resulting in a decreased shoot number. Shoot tips of* A. benthamii* cultured on MS ×1/2 basal medium supplemented with different concentrations of TDZ and IAA/NAA resulted in 20.1 times the average number of multiple shoots. Such results are quite similar to the earlier reports on* Crocus sativus* [2],* Hyoscyamus niger* [26], and* Cichorium intybus* [4, 5], where TDZ was found to be effective in enhancing the shootlet formation. However, further increase in both auxin/cytokinin concentrations was not found to be favorable. Similarly, organogenesis was also noticed from the leaf-derived callus of* Arnebia euchroma* [27] and* Bergenia ciliata* [28]. The additive effect of IAA and TDZ has been seen in numerous plants such as* Santolina canescens* [29] and* Bupleurum fruticosum *[30], which confirms that even the least auxin concentrations in combination with a cytokinin positively tailored the frequency of shoot induction and plant growth. In the present study, the combined interaction of BAP and IBA resulted in maximum multiple shoot regeneration, suggesting that their interaction perhaps resulted in shifts in the endogenous synthesis of TDZ and IAA, making it either suboptimal or supraoptimal and resulting in fulfilling varying needs in the exogenous supply of both hormones [31]. Therefore, in* A. benthamii, *the optimum level of exogenously supplied phytohormones to maximum shoot multiplication has been registered to be 4 *μ*M TDZ and 4.5 *μ*M IAA.

### 3.2. Root Hardening and Field Trial

The elongated and semielongated shoots recorded from the multiplication phase were transferred to MS ×1/2 medium supplemented with different concentrations of IBA/IAA (1.0–10.0 *μ*M) with TDZ (0.5 *μ*M–5.0 *μ*M) for induction and elongation of roots (Table 3). Elongation as well as root initiation was noticed after a 6–8-week culture period. The basal zone of the elongated shoots first turned black and apparently became hard. Lower concentrations of IBA (1–2.0 *μ*M) failed to promote any desired response. However, when the concentration was raised, the basal zone of the shoots became blacker, hard, and elongated, thus giving a tap root-like appearance, followed by the initiation of lateral root initials, and a maximum of 5.3 multiple black short, thick rootlets were recorded from the main black hard tap root such as structures on 8 *μ*M of IBA and TDZ (4 *μ*M). Further increase in concentration again had no effect on rooting. When IBA was replaced with IAA (1.0–10.0 *μ*M), the basal zone of the shoots again became hard, resembling a tap root-like structure in different concentrations of IAA. This combination was fruitful for the root generation with a maximum average number of roots (8.3) noticed on 3 *μ*M of IAA and TDZ (1.5 *μ*M) (Table 3 and Figure 1(d)). However, when the concentration was raised from 3 *μ*M to 5.0 *μ*M, no response was noticed. In some treatments, a friable light brownish callus of various degrees was also recorded at different concentrations. A protocol for the complete plantlets formation under* in vitro* conditions and their successful transfer into the greenhouse has been developed. The cytokinins used alone as well in combination with auxins resulted in certain desirable responses, but the most suitable plant growth regulator was found to be the combination of BAP and IBA and TDZ and IAA where maximum multiple shoots were recorded.

The* in vitro* raised plantlets were successfully transferred and kept in a growth chamber present in the laboratory at the requisite temperature and humidity. Initially, caps of the culture vials were removed and culture vials containing* in vitro* born plantlets of* A. benthamii* were kept in the incubation room for 1 week to slowly reduce the high humidity conditions within the culture vials. After these plantlets were transferred from incubation to normal room conditions where* in vitro* plantlets were deflasked, agar was washed under tap water with the help of mild brushing. The plantlets were then potted in small plastic cups containing an autoclaved sand : soil : peat : vermiculite (1 : 1 : 1 : 1) mixture (Figure 1(e)). The potted plantlets were placed in a growth chamber at a temperature of 20–25°C with humidity of 60–70% for one to two weeks followed by their transfer to a greenhouse chamber. These plantlets were then shifted to the greenhouse and were kept there for more than one month. Then, the plantlets were transferred to a net house. The plantlets were continuously monitored, and they showed a response in their growth and development. The plants started showing growth, which indicated their acclimatization behavior. The number of plants transferred to the greenhouse and then into the field was 35 and 64 in 2013-14 and 2014-15, respectively. The survival percentage of plants in the greenhouse was recorded as 75 and 83%, while in the field the number was approximately 60 and 67%, respectively (Table 4 and Figure 1(f)). The tissue culture technique is being increasingly exploited for clonal multiplication and* in vitro* conservation of valuable germplasm threatened by extinction. An efficient procedure for* in vitro* multiplication is an essential prerequisite for employing* in vitro* techniques for germplasm conservation. The present investigation carried out on shoot tips of* A. benthamii* offers a potentially efficient protocol for mass propagation and conservation of this medicinal herb. There is only one published report on* in vitro *studies of* A. benthamii *[32], and the current study uses extensive trials pertaining to the use of growth hormones in addition to successful hardening of plantlets as well. Rooting was observed from multiple shoots and initiation of lateral roots and maximum 8.3 multiple black short thick rootlets was recorded from the main black hard tap root-like structure on 3 *μ*M of IBA and TDZ (4 *μ*M). In earlier reports on* A. euchroma*, rooting was noticed under the influence of IBA [27, 32, 33], and hence our results are in accordance with these. In the present case, IAA was found to be most effective for rooting compared to IBA and NAA. The efficacy of IAA in rooting may be due to its faster uptake than NAA in the present studies.

**Figure 2:**
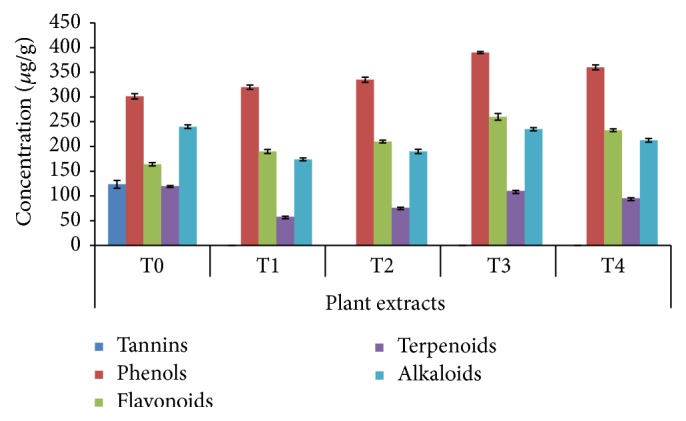
Comparative quantitative estimation of major metabolites present in the* in vitro* plants obtained from various media compositions.

In this experimental line, the four best plant hormonal combinations were selected based on higher shootlet formation. The four selected treatments were assigned as T1 = BAP (4 *μ*M) + IAA (4 *μ*M), T2 = BAP (6.5 *μ*M) + NAA (6 *μ*M), T3 = TDZ (3 *μ*M) + IAA (1.5 *μ*M), and T4 = BAP (5 *μ*M) + NAA (2.5 *μ*M) and were compared with plants (T0 = wild plants) collected from the natural habitat. The results of phytochemical analysis of different types of shoots resulting from application of varieties of media containing various combinations of plant growth regulators (PGRs) are depicted in Figure 2. In the current study, the highest induction of secondary metabolites was observed in T3, obtaining plants with higher amounts of phenols (390 *μ*g/g) followed by flavonoids (260 *μ*g/g) and alkaloids (235 *μ*g/g), which may be due to the influence of the specific plant growth regulator used in the media [34]. PGR has a significant impact on regulation of biosynthetic pathways in plants for synthesis of various metabolites. In addition, the* in vitro* raised plants were recorded to have a higher content of some essential compounds such as phenolic substances, alkaloids, and flavonoids. The results are in line with the findings of various researchers who also report that the PGRs may also influence the production of secondary metabolites under* in vitro* conditions [35, 36]. For example, the accumulation of alkamide and CADs in* Echinacea angustifolia* increased upon addition of cytokines to the culture medium. The amplified biosynthesis of metabolites is allied with tissue differentiation, that is, a clump formation in cell suspensions or the formation of more complex structures [34], which may explain the accumulation of high amounts of metabolites in cultured shoots in our study. However, tannins (123.5 *μ*g/g) and terpenoids (120 *μ*g/g) were found to be higher in T0 plants (Figure 2). The overall discrepancy between the field and* in vitro* plants may be due to a number of factors such as seasonal variation [32], plant-to-plant variation in chemical content, and variation in agroclimatic conditions [37]. It appears to be pertinent to accept the* in vitro* system which may serve as an alternative source of metabolites and thus may be exploited for efficient generation of such substances throughout the year, which are pharmacologically promising but are severely limited in production [14, 38].

### 3.3. Biological Activity

**Figure 3:**
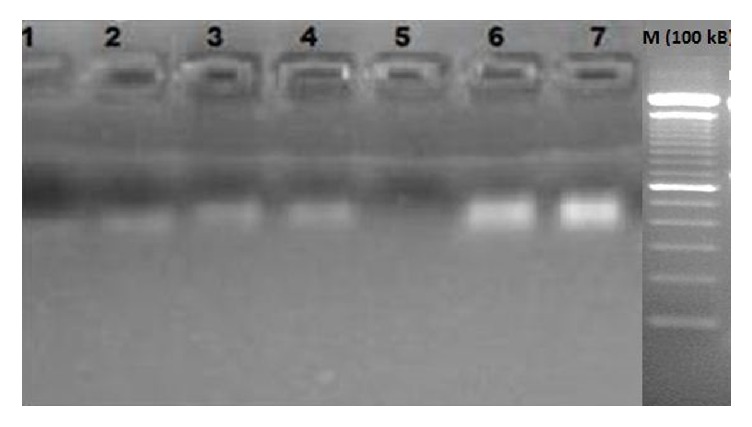
Protective effect of DNA through scavenging of radicals by* A. benthamii* extracts. Lane 1: native calf thymus (ct) DNA + reaction mixture + T1 plant samples (500 *μ*g/ml). Lane 2: native calf thymus (ct) DNA + T2 plant samples (500 *μ*g/ml). Lane 3: native calf thymus (ct) DNA + reaction mixture + T3 plant samples (500 *μ*g/ml). Lane 4: native calf thymus (ct) DNA + reaction mixture + T4 plant samples (500 *μ*g/ml). Lane 5: native calf thymus (ct) DNA + reaction mixture. Lane 6: native calf thymus DNA. Lane 7: native calf thymus DNA + ascorbic acid (500 *μ*g/ml) + reaction mixture.

The oxidative substances completely cause DNA degradation (Figure 3). The scavenging of radicals in different ethyl acetate extracts of* in vitro* raised plants of* Arnebia benthamii* protecting the DNA against damage by ascorbic acid (standard) was determined. In the current study, methanol extracts of all samples obtained from various media combinations under* in vitro* conditions showed significant scavenging of the OH radicals and vis-à-vis protection from DNA damage in ct DNA (Lanes 1–4). The current findings are in continuation of the previous reports on the DNA protective effect of field grown plants of* A. benthamii *[14, 39] suggesting the role of some bioactive compounds in scavenging of hydroxyl radicals [13]. This is the first report depicting the DNA damage protection ability of* in vitro* raised plants, which might be due to the presence of various secondary metabolites as determined above.

## 4. Conclusions

In this study, the first ever-complete protocol for the* in vitro *regeneration and acclimatization under field conditions of* A. benthamii*, a critically endangered medicinal plant of NWH, was developed, leading to a conservation plan for the endangered species. The* in vitro* raised plants, particularly the PGR specific growth, had a significant impact on the presence of volatile/nonvolatile metabolites. The biological efficacy of plant extracts allows their use in drug formulations against possible oxidative DNA damage.

## Figures and Tables

**Table 1:** Effect of GA_3_ enriched MS medium on seed germination of *Arnebia benthamii* Wall.

Growth medium	Germination response	Time period (days) for seed germination initiation	Percentage seed germination response (%)
MS	NR	-	-
MS ×1/2	NR	-	-
MS ×1/2 + GA_3_ (1.5 *µ*M)	NR	-	-
MS ×1/2 + GA_3_ (2.5 *µ*M)	NR	-	-
MS ×1/2 + GA_3_ (5 *µ*M)	Seed germination	45	35
MS ×1/2 + GA_3_ (7.5 *µ*M)	Seed germination	40	35
MS ×1/2 + GA_3_ (10 *µ*M)	Seed germination	40	48
MS ×1/2 + GA_3_ (12.5 *µ*M)	Complete seedling formation	34	53
MS ×1/2 + GA_3_ (15 *µ*M)	Complete seedling formation with elongated shoots	20	78
MS ×1/2 + GA_3_ (17.5 *µ*M)	Complete seedling formation with elongated shoots	30	54
MS ×1/2 + GA_3_ (20 *µ*M)	Seed germination with stunted shoot formation	45	39

*Note.* Data scored after 10 weeks of culture period. NR: no response.

**Table 2:** Effect of different plant growth regulators on shoot multiplication from *in vitro* raised shoot tips of *A. benthamii* on MS ×1/2 medium.

BAP (*µ*M)	TDZ (*µ*M)	IAA (*µ*M)	NAA (*µ*M)	Shoot number	Shoot length (cm)	Callusing (%)	Number of days for minimum shoot formation (*n* = 3)	Minimum shooting response (%)
-	-	-	-	-	-		-	-
1.5		1.0		3.1 ± 1.07^a^	2.0 ± 0.97^a^	+++	55	80
2.5		2.0	-	5.3 ± 0.87^b^	2.3 ± 1.32^a^	+	53	80
*3.5*		3.0	-	8.3 ± 2.9^c^	3.5 ± 1.1^b^	+	44	75
4.5		4.0	-	9.1 ± 2.13^c^	3.4 ± 0.76^b^	+	40	70
5.5		5.0	-	3.2 ± 0.56^ab^	2.1 ± 0.63^a^	+	50	80
6.5		6.0	-	1.1 ± 0.16^a^	2.0 ± 0.87^a^	+++	-	-
*3.5*			3.0	4.5 ± 1.3^b^	2.9 ± 0.13^ab^	+++	50	90
4.5			4.0	8.5 ± 3.2^c^	3.3 ± 1.3^b^	++	45	90
5.5			5.0	11.0 ± 3.1^d^	3.6 ± 0.76^b^	++	45	85
6.5			6.0	12.4 ± 2.6^d^	4.4 ± 1.98^b^	++	35	85
7.5			7.0	11.2 ± 2.1^d^	3.65 ± 2.1^a^	++	30	80
8.5			8.0	9.7 ± 1.4^c^	3.0 ± 1.5^b^	++	46	80
9.5			9.0	5.6 ± 1.65^b^	1.3 ± 0.33^a^	+++	52	90
10.5			10.0	2.2 ± 0.8^a^	1.25 ± 0.78^a^	+++	-	-
	1.0	0.5		9.0 ± 1.8^c^	3.13 ± 1.8^b^	+	40	90
	2.0	1.0		14.2 ± 2.6^d^	3.32 ± 1.32^b^	++	37	100
	*3.0*	1.5		20.1 ± 3.9^e^	5.6 ± 1.6^c^	+	28	100
	4.0	2.0		16.0 ± 2.0^de^	5.3 ± 0.5^c^	+	27	100
	5.0	2.5		12.03 ± 1.4^d^	4.8 ± 0.32^b^	+	35	90
	6.0	3.0		8.9 ± 2.3^c^	3.0 ± 0.97^b^	+	37	90
	7.0	3.5		5.2 ± 1.2^b^	3.0 ± 1.0^b^	+	40	90
	8.0	4.0		3.2 ± 1.6^ab^	2.7 ± 1.43^a^	++	40	90
	*3.0*		1.5	10.5 ± 2.0^cd^	3.0 ± 1.3^b^	++	44	80
	4.0		2.0	13.0 ± 1.5^d^	3.5 ± 1.9^b^	++	35	80
	5.0		2.5	15.3 ± 1.09^d^	4.8 ± 1.5^b^	+	30	90
	6.0		3.0	9.9 ± 2.76^c^	4.4 ± 1.2^b^	++	30	95
	7.0	-	3.5	5.3 ± 1.23^b^	3.1 ± 0.56^b^	++	38	85

*Note.* Data scored after 12 weeks of culture period. Data represented as mean ± SD (*n* = 10); + = low intensity callus formation; ++ = moderate intensity callus formation; +++ = high intensity callus formation. Data was statically analyzed using Duncan multiple range test by SPSS 17.0 software. The values followed by different superscripts are statically significant with each other at *P* < 0.05. TDZ: thidiazuron; IAA: indole 3-acetic acid; Kn: kinetin; BAP: 6-benzylamino purine; IBA: indole 3-butyric acid.

**Table 3:** Effect of different concentrations of auxins (IBA/IAA) with TDZ on root regeneration of *Arnebia benthamii *from multiple shoots.

TDZ (*µ*M)	IBA (*µ*M)	IAA (*µ*M)	Root number	Callusing (%)	Rooting response (%)
1.0	0.5	-	-		-
2.0	1.0	-	-		-
3.0	1.5	-	1.0 ± 0.12^a^	++	70
4.0	2.0	-	1.8 ± 0.55^a^	+++	80
5.0	2.5	-	2.5 ± 0.76^b^	++	80
6.0	3.0	-	2.6 ± 0.3^a^	++	85
7.0	3.5	-	3.7 ± 0.2^bc^	++	85
8.0	4.0	-	5.3 ± 0.0^c^	+	65
1.0	-	0.5	1.9 ± 0.6^ab^	++	80
2.0	-	1.0	3.2 ± 0.5^b^	+++	80
3.0	-	1.5	8.3 ± 0.0^d^	-	85
4.0	-	2.0	7.4 ± 0.0^cd^	-	70
5.0	-	2.5	6.0 ± 0.0^c^	-	80
6.0	-	3.0	3.3 ± 0.0^b^	+	70
7.0	-	3.5	2.12 ± 0.5^b^	+	65
8.0	-	4.0	0.5 ± 0.04^a^	+++	75

Data scored after 12 weeks of culture period. Data represented as mean ± SD (*n* = 10); + = low intensity callus formation; ++ = moderate intensity callus formation; +++ = high intensity callus formation. Data was statically analyzed using Duncan multiple range test by SPSS 17.0 software. The values followed by different superscripts are statically significant with each other at *P* < 0.05. TDZ: thidiazuron; IAA: indol 3-acetic acid; IBA: indole 3-butyric acid.

**Table 4:** Survival percentage of *in vitro* raised plants in greenhouse and field.

Year	Number of plants	Greenhouse (% survival)	^*∗*^Field (% survival)
2013-14	35	75	60
^*∗∗*^2014-15	64	83	67

^*∗*^Values are % of greenhouse survival (%); ^*∗∗*^values are cumulative of 2013 and 2015.
